# Role of Endocrine Gland-Derived Vascular Endothelial Growth Factor (EG-VEGF) and Its Receptors in Adrenocortical Tumors

**DOI:** 10.1007/s12672-015-0236-z

**Published:** 2015-10-16

**Authors:** Dorothee Heck, Sebastian Wortmann, Luitgard Kraus, Cristina L. Ronchi, Richard O. Sinnott, Martin Fassnacht, Silviu Sbiera

**Affiliations:** 1 Department of Internal Medicine I, Endocrine and Diabetes Unit, University Hospital Würzburg, Oberdürrbacher Straße 6, 97080 Würzburg, Germany; 2 Department of Neurology, University Hospital Freiburg, Breisacher Straße 64, 79106 Freiburg, Germany; 3 Central Laboratory, University Hospital Würzburg, Oberdürrbacher Straße 6, 97080 Würzburg, Germany; 4 Department of Computing and Information Systems, University of Melbourne, Parkville, VIC 3010 Melbourne, Australia; 5 Comprehensive Cancer Center Mainfranken, Josef-Schneider-Straße 6, 97080 Würzburg, Germany

**Keywords:** Vascular Endothelial Growth Factor, Merkel Cell Carcinoma, Mitotane, Zona Glomerulosa, Adrenocortical Tumor

## Abstract

Angiogenesis is essential for tumor growth and metastasis. Endocrine gland-derived vascular endothelial growth factor (EG-VEGF) is an angiogenic factor predominantly expressed in steroidogenic organs like the adrenal gland, ovary, testes, and placenta. EG-VEGF has antiapoptotic, mitogenic, and chemoattractive properties mediated via the two G protein-coupled receptors prokineticin receptor 1 (PKR1) and prokineticin receptor 2 (PKR2). We investigated the expression of EG-VEGF and its receptors in a large number of normal adrenal glands (NAG), adrenocortical adenomas (ACA), and carcinomas (ACC) using real-time PCR (NAG, *n* = 12; ACA, *n* = 24; and ACC, *n* = 30) and immunohistochemistry (NAG, *n* = 9; ACA, *n* = 23; and ACC, *n* = 163) and evaluated its impact on patients’ survival. EG-VEGF, PKR1, and PKR2 mRNA and protein are expressed in NAG and the vast majority of ACA and ACC samples. The mean EG-VEGF mRNA expression was significantly lower in ACC (606.5 ± 77.1 copies) compared to NAG (4,043 ± 1,111) and cortisol-producing adenomas (CPA) (4,433 ± 2,378) (*p* < 0.01 and *p* < 0.05, respectively). However, cytoplasmic and nuclear EG-VEGF protein expression was either significantly higher or similar in ACC (*H* score 2.4 ± 0.05, *p* < 0.05 and 1.7 ± 0.08, n.s., respectively) compared to NAG (1.8 ± 0.14 and 1.7 ± 0.2). Nuclear protein expression of either EG-VEGF or PKR1 or both is predictive for a higher mortality compared to patients without nuclear expression (hazard ratio (HR) = 5.15; 95 % confidence interval (CI) = 1.24–21.36, *n* = 100, *p* = 0.02 independent of age, sex, and tumor stage). These findings suggest that EG-VEGF and its receptor PKR1 might play a role in the pathogenesis of adrenocortical tumors and could serve as prognostic markers for this rare malignant disease.

**Electronic supplementary material:**

The online version of this article (doi:10.1007/s12672-015-0236-z) contains supplementary material, which is available to authorized users.

## Introduction

Adrenocortical carcinoma (ACC) is a rare and highly malignant tumor whose pathogenesis is largely unclear [11, 13, 15]. Treatment options are limited and, beside surgery in localized stages, mitotane (adjuvantly or palliatively) or etoposide, doxorubicin, and cisplatin plus mitotane are the current standards [2, 14, 56]. Up to now, only a few prognostic markers are available to guide treatment decisions.

For several decades, it has been established that angiogenesis is essential for tumor growth and metastasis. It is impossible for tumors to expand for more than a few millimeters without neovascularization [21]. Antiangiogenic therapies, mostly targeting the angiogenic key factor vascular endothelial growth factor (VEGF) or its receptor VEGFR-2, are already successfully applied in many solid tumors such as colorectal carcinoma [26], renal cell carcinoma [40], neuroendocrine tumors [48], or thyroid cancer [5, 10, 59]. In 2001, the endocrine gland-derived VEGF (EG-VEGF) was identified as the first tissue-specific angiogenic factor predominantly expressed in steroidogenic organs like the adrenal gland, testes, ovary, and placenta. Both EG-VEGF and VEGF have a HIF-1 binding site and are induced by hypoxia. While sharing mitogenic, permeability enhancing, antiapoptotic, and chemoattractive properties, VEGF and EG-VEGF do not belong to the same gene family [31]. EG-VEGF (also known as prokineticin 1 or PK1) is a secreted glycoprotein and has prokinetic effects on gut [33]. EG-VEGF belongs to the AVIT protein family and shares the amino terminal sequence with prokineticin 2 (mammalian orthologue of Bombina variegata peptide 8), which is not expressed in human adrenal tissue [32]. The two G protein-coupled receptors prokineticin receptor 1 (PKR1) and prokineticin receptor 2 (PKR2) represent cognate receptors for EG-VEGF [36, 38]. EG-VEGF plays a role in the pathology of endocrine tumors, such as Leydig-cell-tumors [51], papillary thyroid cancer [47], and non-endocrine tumors like neuroblastoma [44], prostate cancer [46], gastrointestinal tumors [22, 23, 41, 55], pancreatic ductal adenocarcinoma [27, 39, 49], Merkel cell carcinoma [30], and multiple myeloma [34]. In bovine adrenal cortex-derived endothelial cells, EG-VEGF promotes proliferation, migration, and survival of responsive cells [36].

The adrenal gland is probably the highest vascularized organ in the body [42, 54]. Every adrenocyte is in contact with fenestrated endothelial cells ensuring sufficient oxygenation for hormone biosynthesis [57]. The expression of VEGF in ACC is well examined [1, 8, 29], but very little is known about EG-VEGF in adrenocortical tumors. Thus, we aimed to examine the expression of EG-VEGF, PKR1, and PKR2 in a large number of ACC, adrenocortical adenomas (ACA), and normal adrenal glands (NAG) using real-time PCR (NAG, *n* = 12; ACA, *n* = 24 (cortisol-producing adenoma, *n* = 8; aldosterone-producing adenoma, *n* = 8; endocrine-inactive adenoma, *n* = 8); and ACC, *n* = 30) and immunohistochemistry (NAG, *n* = 9; ACA, *n* = 23 (cortisol-producing adenoma, *n* = 8; aldosterone-producing adenoma, *n* = 8; endocrine-inactive adenoma, *n* = 7); and ACC, *n* = 163). Moreover, we evaluated its relationship with clinical data, including the impact on survival in ACC patients.

## Materials and Methods

### Clinical Data and Specimen

Tissue samples from NAG, ACA, and ACC were collected as described before [17]. Diagnosis was made based on clinical, laboratory, radiological, and pathological results. European Network for the Study of Adrenal Tumors (ENSAT) tumor stage (www.ensat.org) was used for the classification of ACC. Clinical data were collected by the German ACC Registry (www.nebennierenkarzinom.de) and through the European Network for the Study of Adrenal Tumors registry (www.ensat.org). Table 1 displays characteristics of patients and tumors. Patients gave informed consent for collecting tissue and clinical data, and the study was approved by the ethics committee of the University of Wuerzburg (Germany, board approval number 88/11).

**Table 1:** Patients and tumor characteristics

	Age (years)	Sex (M/F)	Tumor size (cm)
Samples used for mRNA expression analysis			
ACC (*n* = 30)^a^	49 (20)	10/18	11.5 (4.0)
ENSAT tumor stage 1 (*n* = 1)	46	0/1	3.0
ENSAT 2 (*n* = 16)	46 (22)	6/10	12.1 (4.0)
ENSAT 3 (*n* = 2)	34 (24)	0/2	12.0 (1.4)
ENSAT 4 (*n* = 9)	59 (12)	4/5	11.4 (3.8)
Endocrine activity (*n* = 23)^b^ Excess of cortisol (+/− other hormones) (*n* = 18) Excess of sex hormones and precursors only (*n* = 2) No hormone excess (*n* = 3)			
NAG (*n* = 12)	53 (10)	8/4	–
ACA (*n* = 24)	52 (15)	12/12	3.5 (2.6)
Cortisol-producing adenoma (*n* = 8)	40 (37)	3/5	2.9 (1.9)
Aldosterone-producing adenoma (*n* = 8)	53 (15)	3/5	1.9 (0.8)
Endocrine-inactive adenoma (*n* = 8)	64 (10)	6/2	5.6 (2.7)
For immunohistochemical analysis			
ACC (*n* = 163)	49 (16)	59/104	12 (4.5)
Primary tumor (*n* = 130)^c^	49 (16)	47/83	12 (4.4)
ENSAT tumor stage 1 (*n* = 5)	54 (24)	2/3	4.7 (0.3)
ENSAT 2 (*n* = 48)	48 (17)	19/29	11.9 (4.5)
ENSAT 3 (*n* = 41)	53 (14)	14/27	11.8 (3.6)
ENSAT 4 (*n* = 34)	47 (18)	11/23	13.4 (4.4)
Endocrine activity (*n* = 84)^d^ Excess of cortisol (+/− other hormones) (*n* = 48) Excess of sex hormones and precursors only (*n* = 13) Excess of mineralocorticoids only (*n* = 4) No hormone excess (*n* = 19)			
Local recurrence (*n* = 19)	46 (17)	9/10	10.6 (3.9)
Metastasis (*n* = 14)	42 (11)	3/11	12 (5.9)
NAG (*n* = 9)	62 (16)	2/7	–
ACA (*n* = 23)	53 (15)	7/16	2.3 (0.7)
Cortisol-producing adenoma (*n* = 8)	45 (12)	0/8	2.8 (0.2)
Aldosterone-producing adenoma (*n* = 8)	46 (12)	3/5	1.7 (0.6)
Endocrine-inactive adenoma (*n* = 7)	70 (5)	4/3	2.2 (0.7)

Data are mean (±SD) or numbers

*ENSAT* European Network for the Study of Adrenal Tumors (www.ensat.org), *M* male, *F* female

^a^In two cases, tumor stage was not determined

^b^No information about hormone production available (5×)

^c^Two patients were lost to follow-up. In two cases, tumor stage was not determined

^d^No information about hormone production available (45×)

### RNA Extraction and Real-Time Quantitative PCR (qPCR)

RNA was extracted from frozen tumor tissue samples (30 ACC, 24 ACA, and 12 NAG) using the RNeasy Mini Kit (Qiagen, Hilden, Germany) according to the manufacturer’s instructions. Reverse transcription was carried out using the iscript TM cDNA Synthesis Kit (Bio-Rad Laboratories GmbH, Munich, Germany) according to the manufacturer’s manual. Samples were diluted with aqua dest in a relation of 1:15 before use as a template.

Real-time quantitative PCR was performed in duplicates using the TaqMan Technology. A reaction mix of 20 μl containing distillated water, TaqMan MasterMix (Applied Biosystems, Darmstadt, Germany), and the primers/probe mixture in the relation 5:10:1 was added to 5 μl of cDNA (original RNA concentration, 3.31 ng/μl). Commercial probes were used (Applied Biosystems, 18s: Hs99999901_s1; EG-VEGF: Hs00951617_m1; PKR1: Hs00373446_m1; PKR2: Hs00431207_m1). A dilution series with a known cDNA copy number allowed absolute quantification of cDNA copy number for each sample.

### Immunohistochemistry in Adrenocortical Tumor Samples

The immunohistochemical stainings were performed on a total of 195 adrenocortical tissue samples (163 ACC, 23 ACA, and nine NAG). The adrenal tumor samples were assembled into three tissue microarrays as described [17, 50]. Immunohistochemical detection was performed using an indirect immunoperoxidase technique following high temperature antigen retrieval in 0.01 M citrate buffer (pH 6.0). As primary antibodies (Table 2), we used EG-VEGF polyclonal rabbit antibody, dilution of 1:200, kindly provided by Elly S. W. Ngan, University of Hong Kong, PKR1 polyclonal rabbit antibody (GPR73A), dilution 1:150, and PKR2 polyclonal rabbit antibody (GPR 73 B), dilution 1:150 (both antibodies from MoBiTec (Molecular Biotechnology), Göttingen, Germany). The signal was developed using the DAKO HRP-System (DAKO, Copenhagen, Denmark) and NovaRed as substrate according to the manufacturer’s instructions (Vector Laboratories, Burlingame, USA). Nuclei were counterstained with hematoxylin. As a negative control, we employed an unspecific IgG isotype antibody and adrenal capsule adipose tissue as an internal control, and as a positive control, we used ovary tissue for EG-VEGF and prostate tissue for PKR1 and PKR2, showing specific cytoplasmatic staining in accordance with www.proteinatlas.org (supplementary Fig. 1). All tissue array slides were analyzed independently by two investigators (D.H. and L.K.). Samples were regarded as evaluable when at least two of five array spots were intact. Where discrepancies were observed, results were double checked by both investigators together with a third observer (S.S.). Cytoplasmic and nuclear staining intensity was assigned to the categories no staining (0), weak (1), moderate (2), and strong (3). The percentage of positive cells was assessed for each specimen and scored 0 if 0 % were positive, 0.5 if 10–49 %, and 1 if 50 % or more cells were positive. A semiquantitative *H* score was calculated by multiplying the staining intensity score with the percentage of positive cells score as described [45]. Later on, for survival analysis purpose, the weak, moderate, and strong stainings were accumulated into a general positive staining.

**Table 2:** Used antibodies, source, and dilution

Protein	Stained protein	Clone/species	Source	Dilution
EG-VEGF	Endocrine gland-derived vascular endothelial growth factor	Polyclonal rabbit	Kindly provided by Elly S. W. Ngan [43]; University of Hong Kong	1:200
PKR1	Prokineticin receptor 1	Polyclonal rabbit	MoBiTec, Göttingen, Germany	1:150
PKR2	Prokineticin receptor 2	Polyclonal rabbit	MoBiTec, Göttingen, Germany	1:150

### Statistical Analysis

Data are presented as mean ± standard error of the mean (SEM). Differences in expression were analyzed using nonparametric Kruskal-Wallis test and Dunn’s post hoc test. Differences in PKR2 mRNA expression were analyzed using nonparametric Mann-Whitney test. We used a Cox regression model for overall survival analyses. Overall survival was defined as time elapsed from primary resection of ACC to death or last follow-up visit. A *p* value < 0.05 was regarded as significant. A univariate and an additional multivariate cox regression analysis including age, sex, and ENSAT tumor stage [12] was carried out. Statistical analyses were performed with SPSS statistics version 22 (SPSS Inc., Chicago, IL, USA) and GraphPad Prism (version 6, GraphPad Software Inc., San Diego, CA, USA).

## Results

### EG-VEGF, PKR1, and PKR2 mRNA Expression

**Fig. 1:**
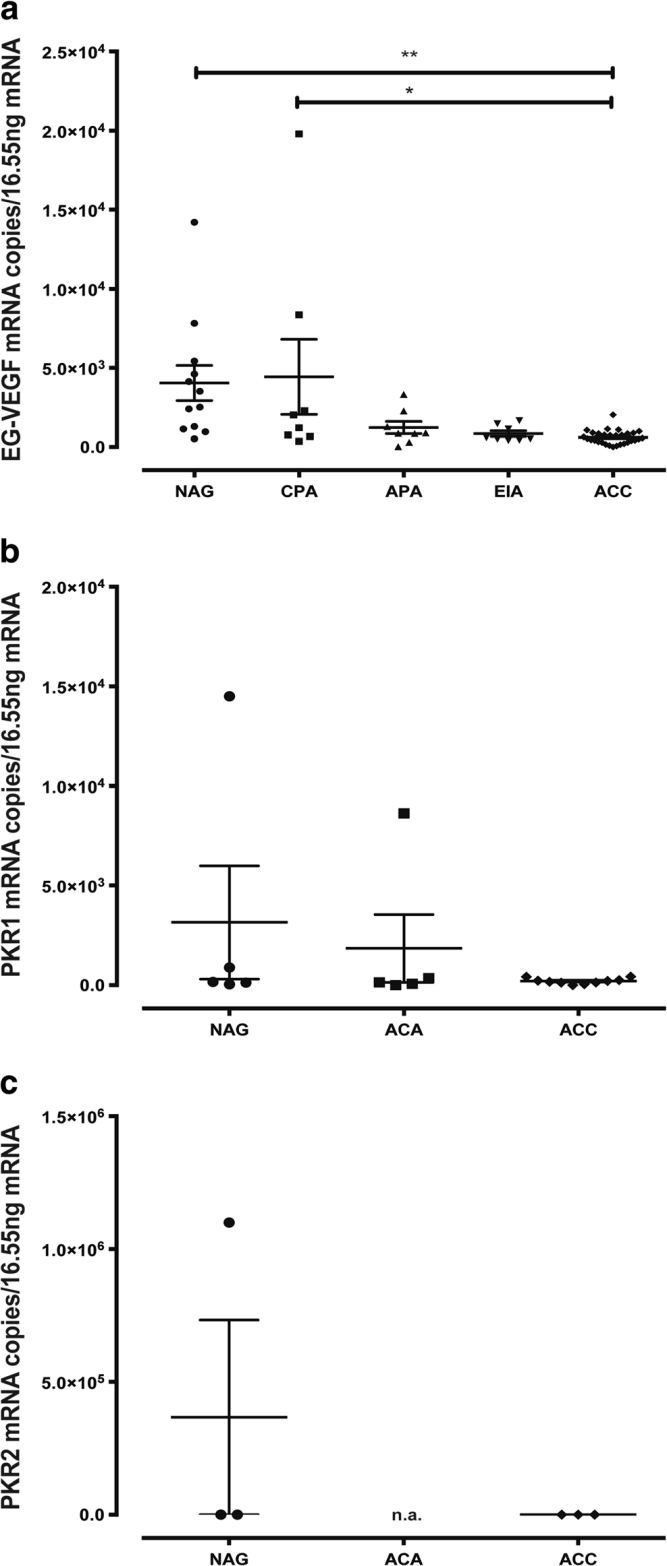
EG-VEGF, PKR1, and PKR2 mRNA expression in adrenal tissues. EG-VEGF (**a**), PKR1 (**b**), and PKR2 (**c**) mRNA copy number/16.55 ng RNA is displayed for every sample. *Black bars* represent means with SEM. *NAG* normal adrenal glands, *ACC* adrenocortical carcinoma, *ACA* adrenocortical adenoma divided in cortisol-producing adenoma (*CPA*), aldosterone-producing adenoma (*APA*), and endocrine-inactive adenoma (*EIA*). *p < 0.05, **p < 0.01

EG-VEGF mRNA was expressed in all NAG (*n* = 12), ACA (*n* = 24), and ACC (*n* = 30). Mean mRNA expression was highest in cortisol-producing adenomas (CPA, 4,433 ± 2,378 copies/16.55 ng RNA) similar to the expression in NAG (4,043 ± 1,111 copies). There was a significantly lower mRNA expression in ACC compared to NAG and CPA (606.5 ± 77 copies, *p* < 0.01 and *p* < 0.05, respectively) using ordinary one-way ANOVA with Turkey’s multiple comparisons test (Fig. 1a). The expression of PKR1 and PKR2 mRNA in NAG, ACA, and ACC was examined on a subset of samples. PKR1 mRNA could be detected in all five NAG (3,148 ± 2,842 copies), nine out of ten ACC (227.8 ± 42.93 copies), and four out of five ACA (two cortisol-producing adenomas, one aldosterone-producing adenoma, one endocrine-inactive adenoma) (2,301 ± 2,110 copies) with the strongest expression in the aldosterone-producing adenoma (8,630 copies) (Fig. 1b). PKR2 mRNA was expressed only very weakly (Fig. 1c).

### EG-VEGF, PKR1, and PKR2 Protein Expression

Specificity of the antibodies was proven using positive and negative controls: The EG-VEGF antibody showed a specific staining on ovary tissue, and the PKR1 and PKR2 antibodies showed a specific staining on prostate tissue. Specific staining was detected in the cytoplasm, not in the nucleus. Employment of an unspecific IgG isotype antibody prevented positive staining, respectively. Moreover, on adrenal capsule tissue, no specific staining was detected (supplementary Fig. 1).

**Fig. 2:**
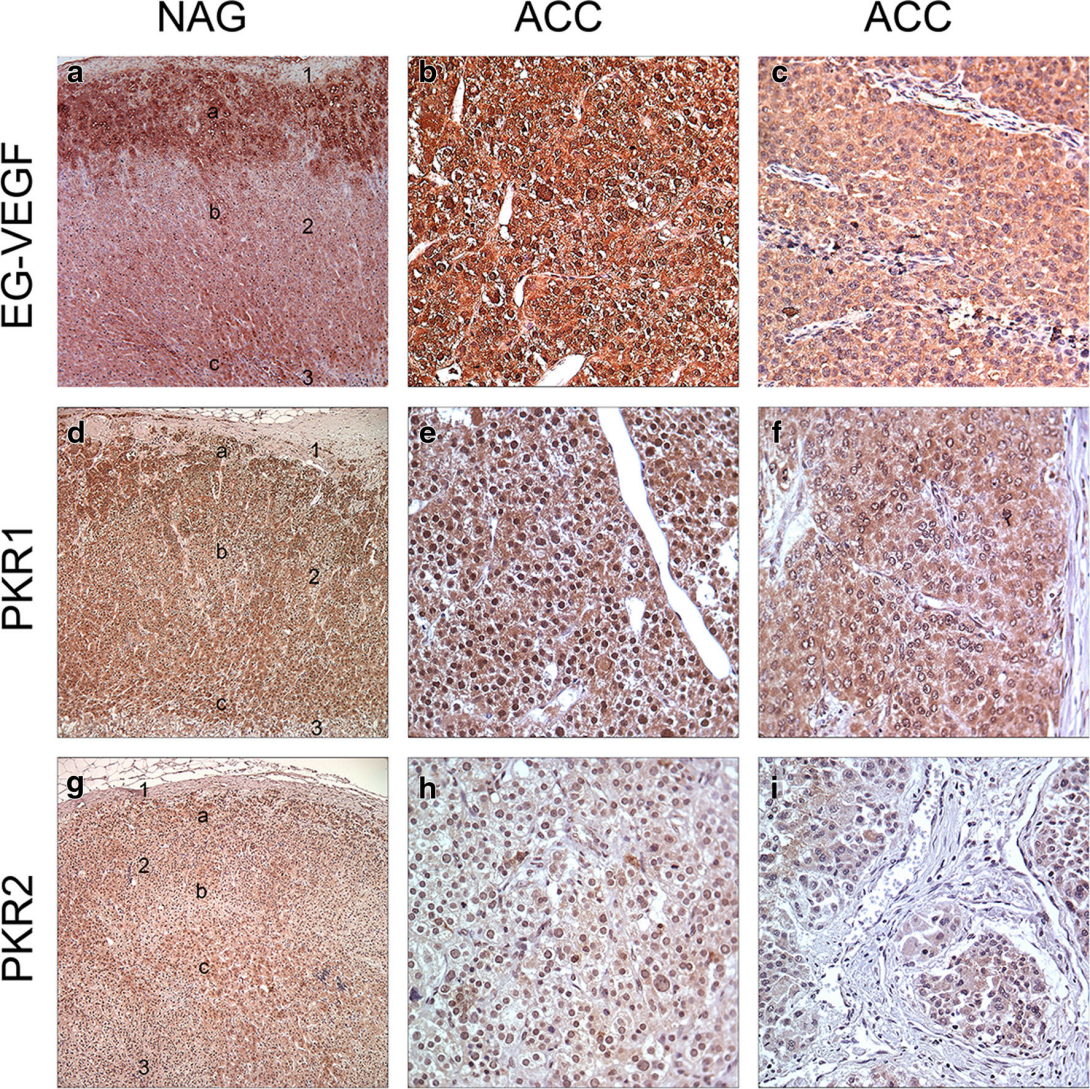
Immunohistochemical staining of normal adrenal glands and adrenocortical carcinoma against EG-VEGF, PKR1, and PKR2. Expression of EG-VEGF (*first row*), PKR1 (*second row*), and PKR2 (*third row*) in normal adrenal glands (**a**, **d**, **g**, *1* = adrenal capsule, *2* = adrenal cortex (*a* = zona glomerulosa, *b* = zona fasciculata, *c* = zona reticularis), *3* = adrenal medulla) and ACC (**b**, **c**, **e**, **f**, **h**, **i**). **b** Example for positive nuclear staining. **c** Example for negative nuclear staining. Magnification: ×40

**Fig. 3:**
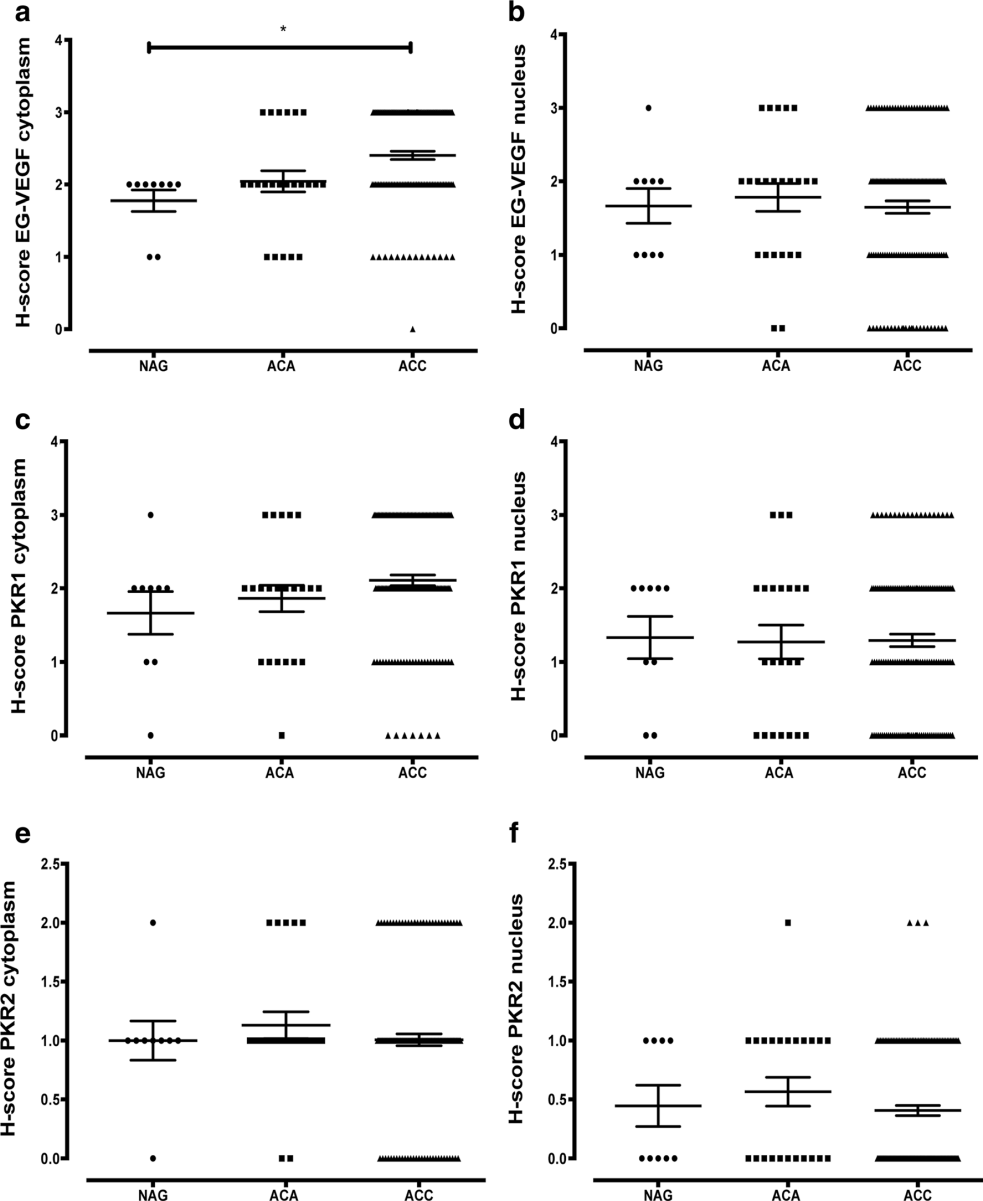
*H* score distribution of EG-VEGF, PKR1, and PKR2 immunohistochemical staining of normal and tumoral adrenocortical tissues. Summary of differential cytoplasmic (**a**, **c**, and **e**) and nuclear (**b**, **d**, and **f**) EG-VEGF (**a** and **b**), PKR1 (**c** and **d**), and PKR2 (**e** and **f**) staining intensity (*H* score) in normal adrenal glands (*NAG*), adrenocortical adenomas (*ACA*), and adrenocortical carcinomas (*ACC*)

The immunohistochemical stainings of NAG, EG-VEGF, PKR1, and PKR2 revealed that these proteins were predominantly expressed in the adrenal cortex and only weakly or absent in the capsule and medulla. EG-VEGF expression was highest in the zona glomerulosa, whereas PKR1 and PKR2 proteins were equally detectable in the zona glomerulosa, zona fasciculata, and zona reticularis. The specific immunostaining was detected both in the nucleus and cytoplasm for all the three investigated proteins (Fig. 2 and Table 3). We therefore decided to evaluate the different cell compartments of each sample. The intra-tumor heterogeneity concerning cytoplasmatic and nuclear staining was predominantly minor (approximately 10 %). Hence, staining intensity and *H* scores were identical. Ninety-nine percent of the evaluable ACC showed a positive cytoplasmic staining against EG-VEGF with 51 % being strong (Table 3). EG-VEGF was also detectable in the cytoplasm of all NAG and ACA (Fig. 3a). Nuclear staining against EG-VEGF was present in 84 % of ACC, 91 % of ACA, and all NAG (Fig. 3b). PKR1 protein was expressed in the cytoplasm of 95 % of ACC, 89 % of NAG, and 95 % of ACA (Fig. 3c). Nuclear staining against PKR1, however, was only observed in 69 % of ACC, 77 % of NAG, and 68 % of ACA (Fig. 3d). In contrast, PKR2 protein staining was either negative or weak to moderate in all samples and independent of subcellular localization (Fig. 3e, f and Table 3). Cytoplasmic EG-VEGF expression was significantly higher in ACC (mean *H* score 2.4 ± 0.06) compared to NAG (mean *H* score 1.8 ± 0.14, *p* < 0.05). Apart from this, nuclear EG-VEGF expression and cytoplasmic and nuclear, PKR1, and PKR2 protein expression did not differ significantly between ACC, NAG, and ACA (Fig. 3).

**Table 3:** Cytoplasmic and nuclear immunohistochemical staining intensity of ACC, adrenal adenomas, and normal adrenal glands against EG-VEGF, PKR1, and PKR2

Staining intensity	Negative	Weak	Moderate	Strong
EG-VEGF cytoplasm				
ACC (*n* = 146)	1 (0.7 %)	14 (9.6 %)	56 (38.4 %)	75 (51.4 %)
Adenoma (*n* = 23)	0	5 (21.7 %)	12 (52.2 %)	6 (26.1 %)
NAG (*n* = 9)	0	2 (22.2 %)	7 (77.8 %)	0
EG-VEGF nucleus				
ACC (*n* = 146)	24 (16.4 %)	35 (24.0 %)	55 (37.7 %)	32 (21.9 %)
Adenoma (*n* = 23)	2 (8.7 %)	6 (26.1 %)	10 (43.5 %)	5 (21.7 %)
NAG (*n* = 9)	0	4 (44.4 %)	4 (44.4 %)	1 (11.1 %)
PKR1 cytoplasm				
ACC (*n* = 137)	7 (5.1 %)	27 (19.7 %)	55 (40.1 %)	48 (35.0 %)
Adenoma (*n* = 22)	1 (4.5 %)	6 (27.3 %)	10 (45.4 %)	5 (22.7 %)
NAG (*n* = 9)	1 (11.1 %)	2 (22.2 %)	5 (55.6 %)	1 (11.1 %)
PKR1 nucleus				
ACC (*n* = 137)	42 (30.7 %)	38 (27.7 %)	47 (34.3 %)	10 (7.3 %)
Adenoma (*n* = 22)	7 (31.8 %)	5 (22.7 %)	7 (31.8 %)	3 (13.6 %)
NAG (*n* = 9)	2 (22.2 %)	2 (22.2 %)	5 (55.6 %)	0
PKR2 cytoplasm				
ACC (*n* = 153)	29 (19.0 %)	94 (61.4 %)	30 (19.6 %)	0
Adenoma (*n* = 23)	2 (8.7 %)	16 (69.6 %)	5 (21.7 %)	0
NAG (*n* = 9)	1 (11.1 %)	7 (77.8 %)	1 (11.1 %)	0
PKR2 nucleus				
ACC (*n* = 153)	94 (61.4 %)	56 (36.6 %)	3 (2.0 %)	0
Adenoma (*n* = 23)	11 (47.8 %)	11 (47.8 %)	1 (4.3 %)	0
NAG (*n* = 9)	5 (55.6 %)	4 (44.4 %)	0	0

Percentages are given in brackets, rounded decimals

### Positive Nuclear Staining Against EG-VEGF and PKR1 Is Predictive for a Higher Mortality

**Fig. 4:**
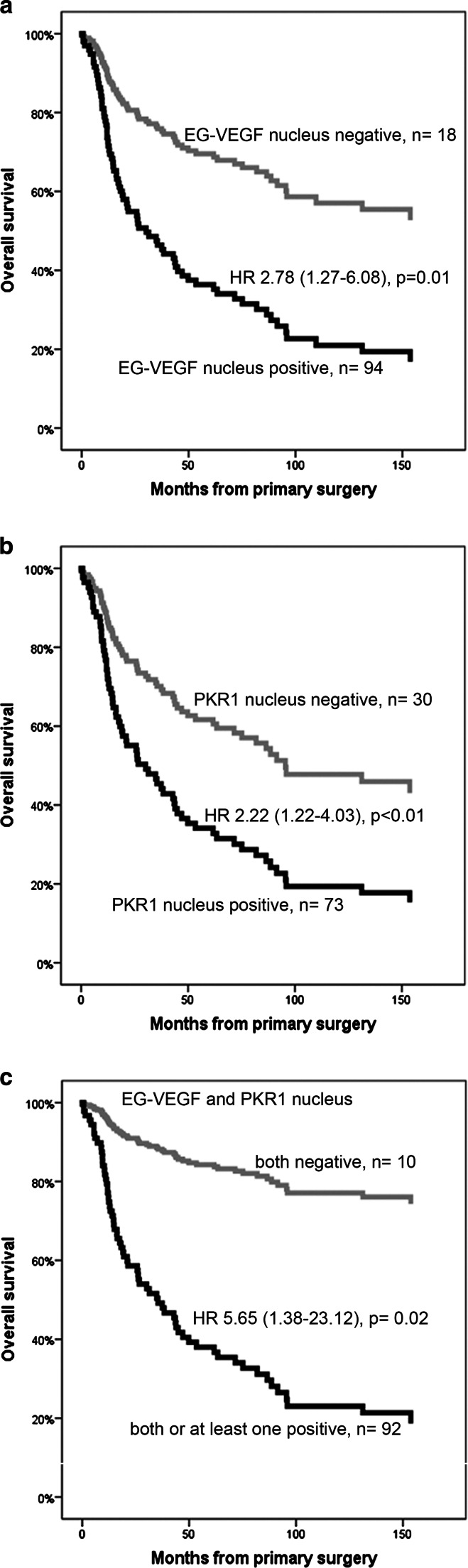
Univariate Cox regression survival curves based on nuclear expression of EG-VEGF and PKR1. **a** Survival of 112 patients with ACC depending on nuclear expression of EG-VEGF: negative nuclear EG-VEGF expression (*grey*) and positive (*black*). **b** Survival of 103 patients with ACC depending on nuclear expression of PKR1: negative nuclear PKR1 expression (*grey*) and positive (*black*). **c** Survival of 102 patients with ACC depending on nuclear expression of EG-VEGF and PKR1: both negative (*grey*) and both or at least one positive (*black*)

We performed overall survival analyses using Cox regression plots only in ACC samples coming from primary tumors with available immunostaining and clinical data (for multivariate analysis: EG-VEGF *n* = 110, PKR1 *n* = 101, PKR2 *n* = 115) (Fig. 4 and Table 4). Cytoplasmic EG-VEGF, PKR1, and PKR2 expression did not correlate with overall survival. However, a positive nuclear staining against EG-VEGF was associated with a significantly higher mortality in patients with ACC (hazard ratio (HR) for death 2.78; 95 % confidence interval (CI) 1.27–6.08; *p* = 0.01) (Fig. 4a). Similarly, patients with a positive nuclear staining against PKR1 were more likely to die compared to patients with a negative nuclear expression of PKR1 (HR 2.22; 95 % CI 1.23–4.03; *p* < 0.01) (Fig. 4b). The prognostic value was even higher when either EG-VEGF or PKR1 protein or both were expressed in the nucleus of ACC cells compared to patients with none of these factors in the nucleus (HR 5.65; 95 % CI 1.38–23.12; *p* = 0.02) (Fig. 4c). Multivariate regression analysis including age, sex, and ENSAT tumor stage confirmed the independent prognostic value of this combination (EG-VEGF: HR 2.41, 95 % CI 1.08–5.38, *p* = 0.03; PKR1: HR 1.95, 95 % CI 1.06–3.56, *p* = 0.03; EG-VEGF or PKR1: HR 5.15, 95 % CI 1.24–21.36, *p* = 0.02) (Table 4). Excess of cortisol production did not influence survival in our patients (HR 1.06, CI 0.63–1.76, *p* = 0.84) (supplementary Fig. 2).

**Table 4:** Factors influencing overall survival in patients with ACC according to univariate and multivariate analysis

Variables	Univariate analysis			Multivariate analysis^a^		
	HR	95 % CI	*p*	HR	95 % CI	*p*
Age						
Younger than median (*n* = 64)^b^						
Older than median (*n* = 64)	1.34	0.88–2.05	0.18	1.38	0.89–2.13	0.15
Sex						
Male (*n* = 46)^c^						
Female (*n* = 82)	0.95	0.62–1.48	0.83	0.84	0.53–1.31	0.43
ENSAT tumor stage						
I–II (*n* = 53)^d^						
III (*n* = 39)	1.79	1.05–3.06	0.03	1.78	1.04–3.06	0.04
IV (*n* = 34)	4.14	2.39–7.19	<0.001	4.25	2.44–7.41	<0.001
EG-VEGF cytoplasm						
Negative + weak (*n* = 9)^e^						
Moderate + strong (*n* = 103)	2.59	0.82–8.25	0.11	2.00	0.60–6.33	0.27
EG-VEGF nucleus						
Negative (*n* = 18)^d^						
Positive (*n* = 94)	2.78	1.27–6.08	0.01	2.41	1.08–5.38	0.03
PKR1 cytoplasm						
Negative (*n* = 5)^d^						
Positive (*n* = 98)	1.71	0.54–5.46	0.37	2.33	0.71–7.66	0.16
PKR1 nucleus						
Negative (*n* = 30)^d^						
Positive (*n* = 73)	2.22	1.22–4.03	0.01	1.95	1.06–3.56	0.03
PKR2 cytoplasm						
Negative (*n* = 19)^d^						
Positive (*n* = 98)	1.57	0.83–2.97	0.17	1.58	0.82–3.04	0.17
PKR2 nucleus						
Negative (*n* = 69)^d^						
Positive (*n* = 48)	1.13	0.72–1.78	0.59	1.33	0.84–2.13	0.23
EG-VEGF and PKR1 nucleus						
Both negative (*n* = 10)^d^						
Both or at least one positive (*n* = 92)	5.65	1.38–23.12	0.02	5.15	1.24–21.36	0.02

Only primary tumor samples were used for this survival analysis. Two patients were lost to follow-up. Samples were not evaluable, if less than two of five spots were intact (EG-VEGF array, 16; PKR1 array, 25; PKR2 array, 11). Therefore, the number of samples is slightly different from Tables 1 and 3

*HR* hazard ratio, *95 % CI* 95 % confidence interval

^a^The multivariate analysis included age, sex, and tumor stage (three groups, owing to the low number of patients with ENSAT tumor stage 1, these were combined with patients with ENSAT tumor stage 2 into one group) as covariates. In two cases, tumor stage was not determined

^b^Younger age than the median was taken as the reference category

^c^Male sex was the reference category

^d^ENSAT stage I and II was the reference category

^e^Since cytoplasmic staining against EG-VEGF was negative in only one probe, negative and weak staining were combined as the reference category

^d^Negative staining was the reference category

## Discussion

The key finding of our study is the strong prognostic potential of nuclear staining of EG-VEGF and its receptor PKR1 for patient outcome in ACC. In general, EG-VEGF and both of its receptors PKR1 and PKR2 are present in most adrenocortical adenomas and carcinomas. However, only the nuclear localization harbors prognostic value. Indeed, we are the first to describe nuclear expression of the glycoprotein EG-VEGF and its G protein-coupled receptors PKR1 and PKR2. The specificity of our antibodies was proven on negative controls and positive controls in accordance with www.proteinatlas.org and previous publications on the expression of EG-VEGF in ovary tissue [18, 20] and PKR1 and PKR2 in prostate tissue [46]. The normal tissues showed in contrast to tumor tissues no nuclear staining. Over the last years, knowledge about intracellular protein transport has increased. It has been shown that subcellular trafficking of proteins to the “wrong” cell compartment, such as the nucleus for a membrane receptor, can result in disease like cancer by loss of function or gain of activity in the “wrong” cell compartment. Such nuclear misleading of proteins is known for epidermal growth factor receptor (EGFR) [4], FGF [37], and VEGF receptor [16]. Concerning EG-VEGF, this nuclear expression might represent active interaction of both ligand and receptor required for relevant influence on cell cycle and transcription. Remarkably, the EG-VEGF promotor has a potential binding site for an orphan nuclear receptor essential for adrenal development, steroidogenic-factor 1 (SF)-1, or NR5A1 [32]. In neuroblastoma cells, bovine adrenal cortex capillary endothelial cells and bovine glomerulosa and fasciculata cells, an autocrine proliferation mechanism of EG-VEGF, probably via its receptor PKR1, could be demonstrated [28, 35, 44]. Thus, we hypothesize that there exists a similar mechanism in tumor growth in ACC. Of note, if either EG-VEGF or PKR1 or both of them are present in the nucleus, the likelihood that patients die from ACC is more than five times higher than if none of these factors are detectable. Interestingly, this result is exactly confirmed in multivariate analysis.

Up to now, only the tumor stage is generally accepted as a prognostic tool. However, within a given tumor stage, survival of ACC patients is quite heterogeneous [25] resulting in uncertainty of clinicians regarding aggressiveness of treatment when confronted with an individual patient. Therefore, reliable prognostic markers are urgently needed. In the last years, few immunohistochemical markers with prognostic potential have been suggested [3, 9, 17, 52, 53, 58]. In addition to these markers, nuclear staining of EG-VEGF and its receptor PKR1 is interesting owing to its high prognostic value.

To our knowledge, this is the first report of the expression of EG-VEGF and its receptors PKR1 and PKR2 in a large number of NAG, ACA, and ACC. In our small mRNA study, EG-VEGF mRNA expression was significantly higher in NAG compared to ACC. Conversely, cytoplasmic EG-VEGF protein expression was significantly higher in ACC compared to NAG. There are multiple reasons for this discrepancy in mRNA and protein expression: Apart from the possible inaccuracy of technical methods and low sample size (only eight patients were identical in mRNA and protein analysis), mRNA expression does not always predict protein expression, especially in genes involved in development and regulation. Alternative splicing, translational modifications, and different degradation of mRNA and protein all have an impact on mRNA and protein quantities [24]. Besides the significant different EG-VEGF mRNA and cytoplasmic expression in ACC and NAG, the mRNA and protein expression of EG-VEGF, PKR1, and PKR2 did not show any significant differences between the adrenal entities. A possible explanation might be the existing strong vascularization of the normal adrenal gland as an endocrine organ [57], which is still present in adrenocortical tumors, although the vascular density in ACC is relatively lower than in NAG and ACA [1]. Moreover, angiogenesis is a highly complex process requiring the precise coordination of many angiogenic factors. Physiologic and pathologic angiogenesis are still not fully understood [6, 7, 19]. It is conceivable that the angiogenic factors EG-VEGF and VEGF interact in the adrenal gland as assumed by Thomas et al. [57].

The expression pattern of EG-VEGF, PKR1, and PKR2 protein in the adrenal cortex of NAG was different in human tissue compared to previous examinations by Keramidas et al. on bovine adrenal cortex tissue [28]. Both in human and bovine tissues, EG-VEGF, PKR1, and PKR2 were predominantly detectable in the cortex with only very weak, respectively, no, specific staining in the medulla or adrenal capsule. EG-VEGF expression was highest in the zona glomerulosa in human tissue, whereas EG-VEGF staining was slightly stronger in the zona fasciculata/reticularis in bovine tissue. PKR1 and PKR2 expression also differed among the two species with regard to a stronger expression of PKR2 in the bovine zona glomerulosa. A different expression pattern between the two species is in accordance with a previous study indicating a different expression of EG-VEGF among mammalian species, probably due to divergence in the promoter sequence [32].

Furthermore, EG-VEGF would be an interesting target for antiangiogenic therapies against ACC or tumors of the ovary and testes. In contrast to widespread VEGF, against which antiangiogenic therapies have already been successfully established, EG-VEGF is predominantly expressed in steroidogenic organs. Therefore, fewer side effects would be expected in anti-EG-VEGF therapies in comparison to anti-VEGF therapies. However, anti-EG-VEGF therapies might possibly cause gastrointestinal side effects given its prokinetic effect on gastrointestinal small muscle.

In summary, our work suggests an implication of EG-VEGF and its receptor PKR1 in pathogenesis of ACC. However, the most important finding is that nuclear staining of EG-VEGF together with PKR1 is one of the best prognostic markers for overall survival in patients with ACC.

## Electronic Supplementary Material

Below is the link to the electronic supplementary material.

Supplementary Fig. 1 Positive and negative controls for EG-VEGF-, PKR1-, and PKR2-antibodies. Displayed are the positive (**a**, **c**, **e**) and negative controls (**b**, **d**, **f**) for EG-VEGF, PKR1, and PKR2-antibodies, respectively. **A**: ovary tissue showing specific cytoplasmatic staining against EG-VEGF. **C**: prostate tissue showing specific cytoplasmatic staining against PKR1. **E**: prostate tissue showing specific cytoplasmatic staining against PKR2. **B**, **D**, **F**: negative controls with employment of an unspecific IgG isotype antibody to **B**: ovary tissue, **D** and **F**: prostate tissue. Magnification: ×10, small boxes ×40. (JPEG 2,327 kb)

Supplementary Fig. 2 Univariate Cox regression survival curves based on excess cortisol production. Survival of 84 patients with ACC depending on excess cortisol production (+/− other hormones) (*black*), *n* = 48, and no excess of cortisol (*grey*), *n* = 36. (JPEG 552 kb)
