# Supplementary figures and images for: Role of Endocrine Gland-Derived Vascular Endothelial Growth Factor (EG-VEGF) and Its Receptors in Adrenocortical Tumors
Source: Horm Cancer. 2015 Oct 16;6(5-6):225–36. doi: 10.1007/s12672-015-0236-z (PMC4630243; doi:10.1007/s12672-015-0236-z)

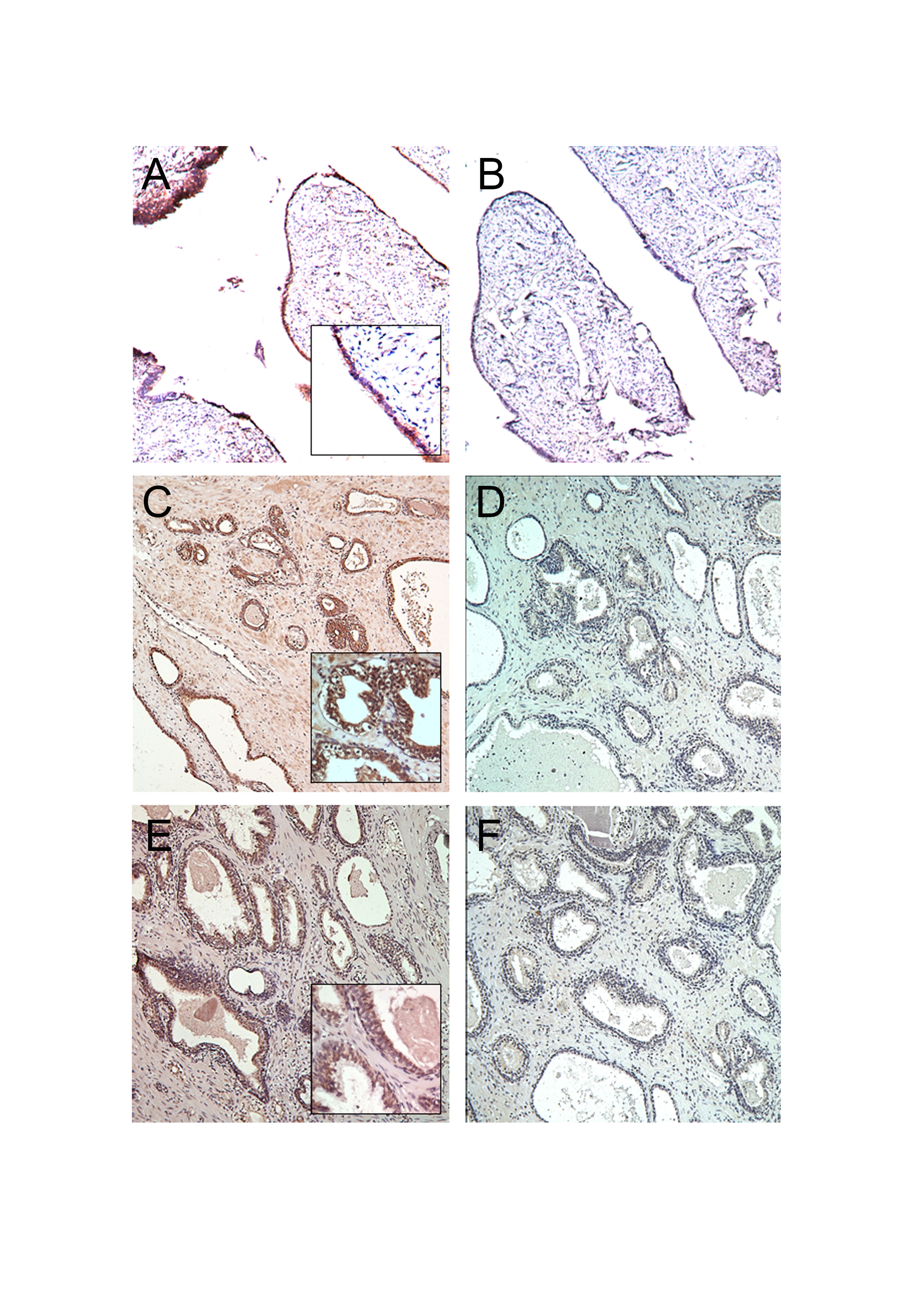

Supplement: Supplementary file 1 — Positive and negative controls for EG-VEGF-, PKR1-, and PKR2-antibodies. Displayed are the positive (a, c, e) and negative controls (b, d, f) for EG-VEGF, PKR1, and PKR2-antibodies, respectively. A: ovary tissue showing specific cytoplasmatic staining against EG-VEGF. C: prostate tissue showing specific cytoplasmatic staining against PKR1. E: prostate tissue showing specific cytoplasmatic staining against PKR2. B, D, F: negative controls with employment of an unspecific IgG isotype antibody to B: ovary tissue, D and F: prostate tissue. Magnification: ×10, small boxes ×40. (JPEG 2,327 kb) [file 12672_2015_236_MOESM1_ESM.jpg]

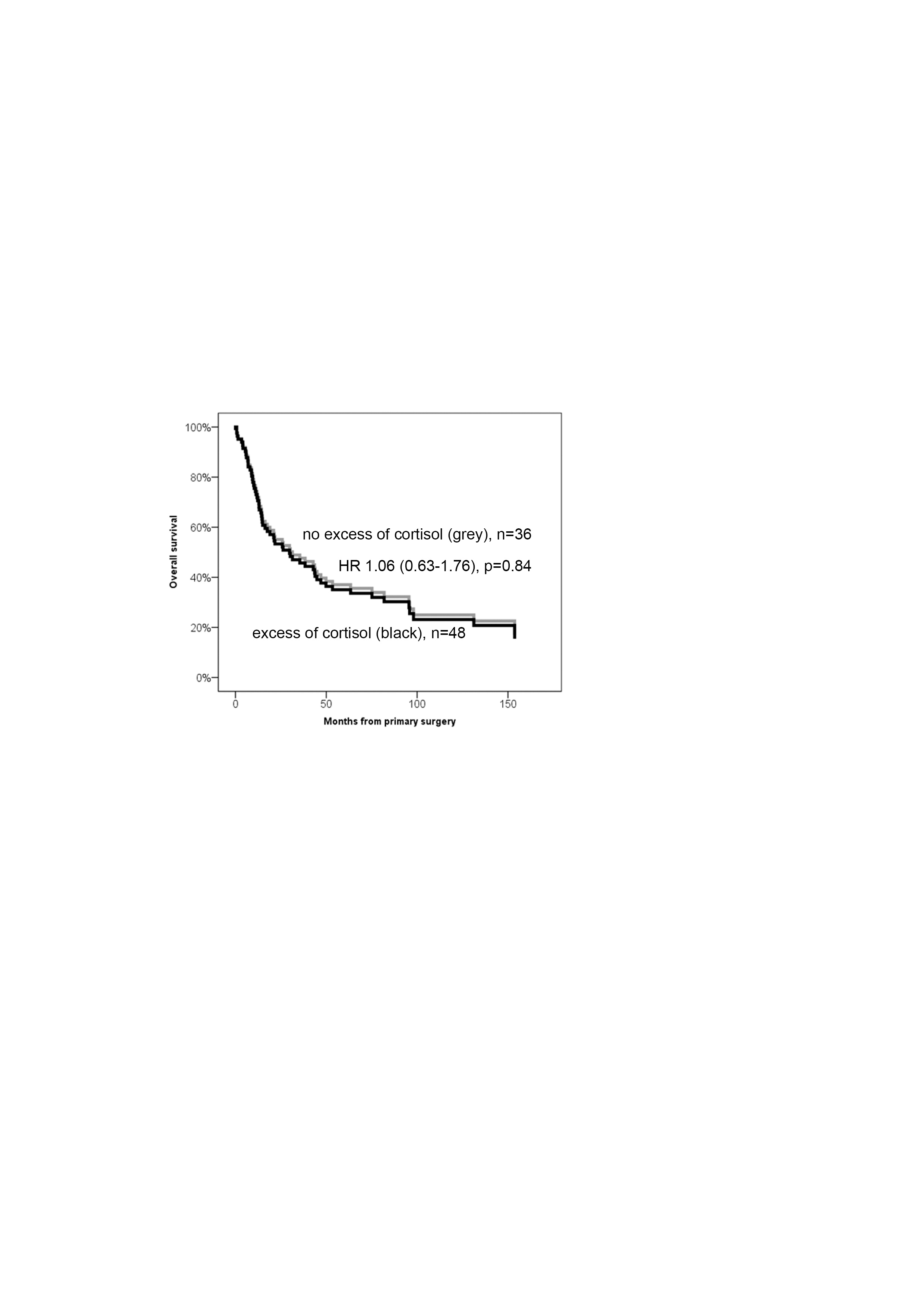

Supplement: Supplementary file 2 — Univariate Cox regression survival curves based on excess cortisol production. Survival of 84 patients with ACC depending on excess cortisol production (+/− other hormones) (black), n = 48, and no excess of cortisol (grey), n = 36. (JPEG 552 kb) [file 12672_2015_236_MOESM2_ESM.jpg]
